# Assessing the Utility of a Novel SMS- and Phone-Based System for Blood Pressure Control in Hypertensive Patients: Feasibility Study

**DOI:** 10.2196/cardio.7915

**Published:** 2017-07-27

**Authors:** Robert Mattson Peters, Nishkala Shivakumar, Ran Xu, Kavon Javaherian, Eric Sink, Kunjan Patel, Angela Brown, Justin Huynh, Melvin Blanchard, Will Ross, Jonathan Byrd

**Affiliations:** ^1^ Washington University in St. Louis School of Medicine St. Louis, MO United States; ^2^ Saint Louis University School of Medicine St. Louis, MO United States; ^3^ Mercy Clinic St. Louis, MO United States

**Keywords:** telemedicine, hypertension, quality improvement, text messaging, primary care, eHealth, mHealth, disease management

## Abstract

**Background:**

Although hypertension (HTN) is a major modifiable risk factor for arterial damage, blood pressure (BP) remains poorly controlled in the hypertensive population. Telemedicine is a promising adjunct intervention that may complement traditional therapies and improve adherence rates; however, current approaches have multiple barriers to entry, including the use of relatively expensive Bluetooth devices or the dependence on smart phone utilization, which tend to exclude low-income and more elderly populations.

**Objective:**

The aim of this study was to design and implement a new phone call- and short message service text messaging-based intervention, Epharmix’s EpxHypertension, in a quality improvement project that demonstrates the feasibility of this system for BP control in a family medicine setting.

**Methods:**

We recruited 174 patients from a community clinic in St Louis from a database of patients diagnosed with HTN. An automated call or text messaging system was used to monitor patient-reported BPs. If determined to be elevated, physicians were notified by an email, text, or electronic medical record alert. Mean systolic BPs (SBPs) and diastolic BPs (DBPs) were compared at the beginning and end of 12 weeks.

**Results:**

After 12 weeks on the system, patients with a baseline SBP of 140 mm Hg or higher reduced SBP by 10.8 mm Hg (95% CI −14.5 to −7.2, *P*<.001) and DBP by 6.6 mm Hg (95% CI −9.9 to −3.4, *P*=.002), but no significant changes were observed in overall BPs and BPs in the group with baseline SBP less than 140 mm Hg.

**Conclusions:**

EpxHypertension provides a viable means to control HTN in patients with high baseline BPs despite previous therapy. This community implementation study demonstrates the feasibility of implementing EpxHypertension across a primary care setting without the need for smartphones or Bluetooth-linked BP cuffs. Future studies should evaluate its effectiveness in a randomized control trial compared with standard of care.

## Introduction

Hypertension (HTN) is a major risk factor for cardiovascular events such as stroke, heart failure, and myocardial infarctions [1]. Over 85.7 million adults in the United States have HTN [2]. In 2013, the estimated direct and indirect cost of HTN was US $51.2 billion [2]. Although lowering blood pressure (BP) has been shown to improve outcomes [3,4], only 54% of hypertensive patients in the United States are considered to have controlled HTN [5]. In strategies to manage BP, self-monitoring has been shown to predict health outcomes better than office BP measurements [6,7] and lead to a lowering of BP over time [8]. However, manual BP logs that are typically used to record at-home measurements [8,9] are often lost or not adequately utilized for clinical management.

Telemedicine has been studied as a promising and efficacious way to improve health outcomes across many conditions, including HTN [10-13]. Many tools have utilized smartphone apps or Internet-linked BP cuffs [14-17], but several studies have experienced barriers such as connectivity issues, low health literacy, and high cost while using these technologies [16,18]. Significant overhead cost is a limiting factor for utilization in socioeconomically disadvantaged populations and prohibits widespread use in the general population. For example, Internet or Bluetooth-linked cuffs can be up to tenfold more expensive than nonlinked cuffs and require patients to use a smartphone device to directly connect. These higher technological requirements also tend to discriminate against elderly patients.

Utilizing short message service (SMS) text messaging is a potential solution to some of these obstacles because of its high accessibility: 86% of American adults who earn less than $30,000 in a year own a cell phone [19]. Studies have shown that SMS usage can increase treatment compliance, including medication adherence [20]. Various telemedicine HTN interventions have shown some success in improving health outcomes, including call-based [21] and SMS-based [22] interventions. Despite the recent developments of telemedicine interventions for HTN, the feasibility of combined text messaging and phone calls without the need for Internet-linked platforms for HTN management has not been extensively studied [23]. Although there are SMS-based systems with Internet-linked platforms that have demonstrated BP management, there are no telemedicine interventions that have found significant reductions in BP using a combination of phone call and text messaging for patients with nonlinked cuffs.

We hypothesized that patients would be willing and able to both regularly measure and manually report their BP along with important contextual information via automated phone calls and text messages, thereby expanding the population who can benefit from mobile health (mHealth) solutions. To test this question, we utilized Epharmix, an automated calling and text messaging platform that sends standardized condition-specific messages to patients and their health care providers to track symptoms longitudinally in real time, provides educational content to patients, and triggers alerts to providers when patients report concerning symptoms or behaviors. In this study, we developed and examined the utilization and effect of a BP monitoring system, EpxHypertension, a phone and SMS text messaging system for patients with nonlinked cuffs. We hypothesized that providing clinicians with real-time data would allow them to appropriately intervene for patients exhibiting poorly controlled HTN, thereby leading to improved BP control for the patients enrolled in EpxHypertension.

## Methods

Our quality improvement study was designed to evaluate the feasibility of the EpxHypertension system for BP control. A list of patients with a documented diagnosis of HTN via the International Statistical Classification of Diseases and Related Health Problems, Ninth Revision (ICD-9) and ICD-10 code was created from a community clinic in St Louis, Missouri. Participants were enrolled from June 27, 2016 to September 10, 2016 and followed for 12 weeks. Assistants called each patient to explain the intervention and offer enrollment pursuant to institutional policies. We contacted 353 patients, and 174 patients consented to being enrolled in EpxHypertension. The consenting patients were then enrolled in either the text messaging (58.6%, 102/174 patients) or phone call (41%, 72/74 patients) system according to their preference. For patients who were unable to text for technological or personal preference, an identical automated phone call was sent following the same algorithm as the text messages. Eligible patients were above the age of 18 years, had access to a phone, and owned an at-home ambulatory BP monitor. No exclusions were made based on the type of monitor (wrist vs upper arm), and no additional instruction was given on how to measure BP. To design an effectiveness study, it was impractical to control the exact type of BP monitor being used across the population, especially as implemented at this scale. However, this did add the limitation of adding more potential noise from inaccurate results. Of the 174 patients who consented, 44 never responded to the initial automated message or phone call sequence and were not sent any future messages or calls. A total of 105 patients had completed through to week 12 of the study at the time of analysis.

For the first 2 weeks, patients were asked to measure and report their BP to the automated system on a daily basis, at the same time each day, with instructions to measure their BP after sitting for at least 5 min if they had recently been physically active. Baseline was defined as the mean of the first 5 responses within the first 2 weeks. After 2 weeks, the system’s dynamic scheduling algorithm adjusted message frequency based on BP control. If baseline systolic BP (SBP) was 140 mm Hg or higher or diastolic BP (DBP) was 110 mm Hg or higher, texts or calls were sent daily asking for self-reported BP values. If baseline SBP was less than 140 mm Hg and DBP was less than 110 mm Hg, texts were sent 3 times per week. Our smart schedule system also set message frequency to 3 times a week if a patient’s most recent bimonthly (every 2 weeks) average SBP and DBP were less than 140 mm Hg and 100 mm Hg, respectively. Message frequency was increased to daily if a patient’s most recent bimonthly average SBP and DBP were greater than/equal to 140 mm Hg and 100 mm Hg, respectively.

The system automatically alerted a physician if the patient’s SBP was outside the threshold range (system default or set by the provider), and the patient was prompted to contact his or her provider immediately. The default threshold range set by the system for SBP was between 90 and 180 mm Hg and between 60 and 110 mm Hg for DBP for one-time measurements, along with a bimonthly mean DBP of more than 100 mm Hg. These thresholds were made based on the recommendations by the American Heart Association (AHA), which designate high BP to be 140/90 mm Hg or higher and a hypertensive urgency to be 180/110 mm Hg or higher [20]. The threshold range could be modified by the provider for each patient, if necessary. The physician was recommended to call patients back as he or she deemed necessary within 2 weeks of receiving the alert and utilize the data at follow-up appointments. The messages, however, were left to the provider’s discretion and followed standard HTN management protocols as defined by the AHA. For longitudinal monitoring, providers also received a triaged bimonthly report prioritized based on each patient’s average BP values.

Aggregate deidentified data were provided by Epharmix for analysis. We analyzed 12 weeks of BP data for average change in BP, response rate, and number of hypotensive and hypertensive events (defined as SBP or DBP outside the threshold range). Comparison between the beginning and end of the study period were made using paired *t*-tests (Microsoft Excel 2016) and included patients with at least 5 baseline measurements and at least 2 final measurements. Additional analysis on patient response rate was performed using unpaired *t*-test, Pearson’s correlation, and Fisher’s exact test (Microsoft Excel 2016 and Graphpad Prism). In the analysis, patients were risk stratified based on SBP into two categories: baseline greater than/equal to and less than 140 mm Hg. Automated monthly satisfaction questionnaires were also administered via automated text message or phone call. Patients were asked to assess numerically (Likert-type scale) the overall quality of care, quality of communication with their provider, and satisfaction with frequency of messages or calls. Patients were also able to provide qualitative feedback in the form of text or recorded voice messages in the same survey.

**Figure 1:**
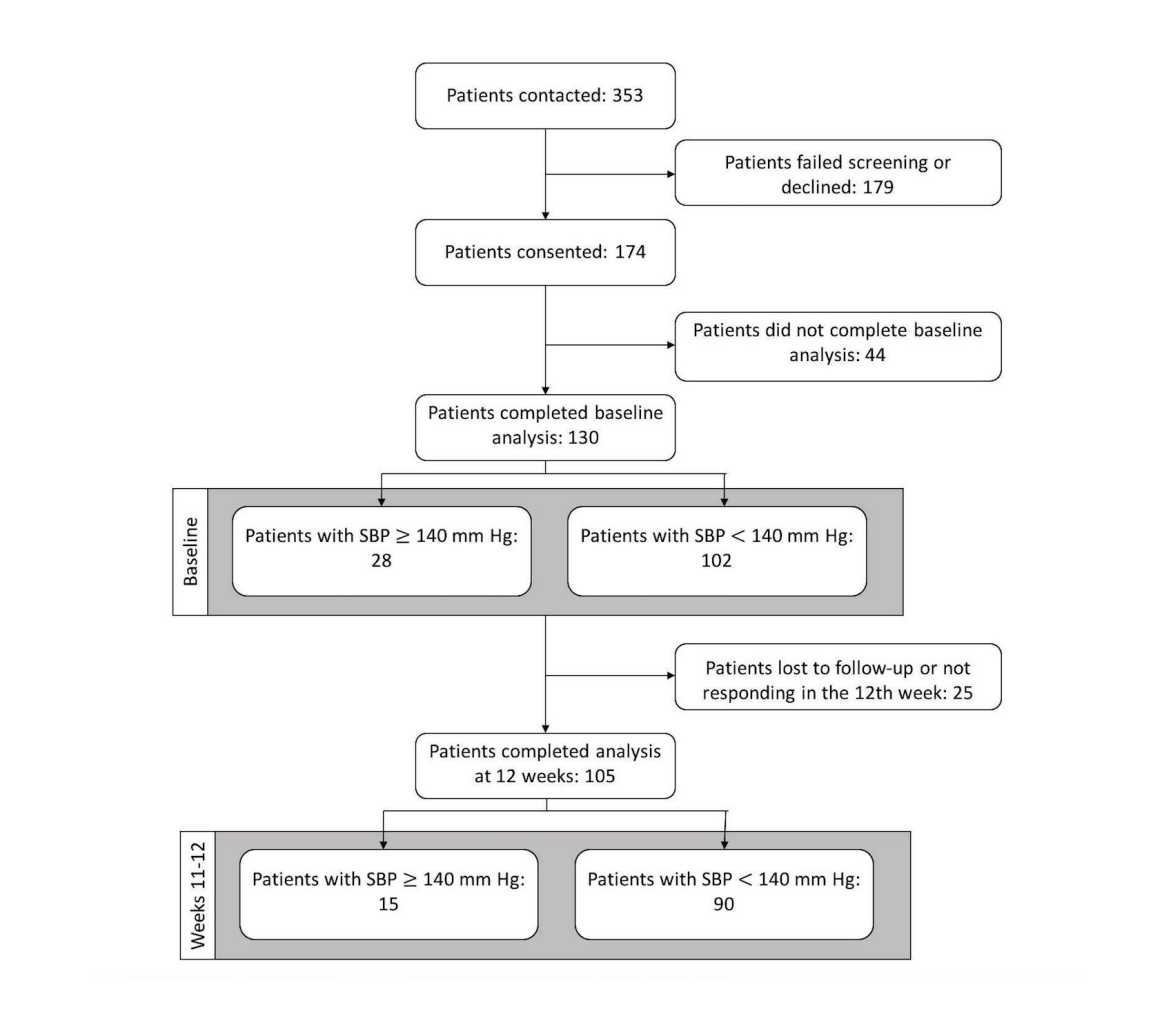
Study flow diagram detailing the stages of the implementation and the number of patients.

## Results

### Response Rate

At the end of the enrollment period, 174 patients diagnosed with HTN were enrolled (Figure 1). In 12 weeks, we received responses for 4781 of the 7345 (65.09%) sent messages and detected 31 events of self-reported SBP and 0 events of self-reported DBP outside the threshold range. Providers were notified about the generated alerts and responded based on their clinical judgment. Of the 130 patients who completed baseline analysis, 105 patients completed the final analysis at week 12. Figure 1 also shows the proportion of enrolled patients reporting SBPs of greater than/equal to and less than 140 mm Hg at baseline and week 12.

Excluding patients who did not complete baseline analysis (44/174), the total response rate was 65.78% (3193/4854 messages). The group with baseline SBP of 140 mm Hg or higher had an average response rate of 62.72% (969/1545 messages), whereas the lower baseline SBP group had an average response rate of 67.21% (2224/3309 messages, *P*=.22). Although there was no significant correlation between change in SBP and response rate, a greater proportion of patients with a response rate of 80% or higher achieved a BP of less than 140 mm Hg by the end of 12 weeks: only 9% (5/58) of patients with a response rate of 80% or higher had an SBP greater than/equal to 140 mm Hg at weeks 11 to 12, compared with 21% (10/47) of patients with a response rate of less than 80% (odds ratio 2.9, 95% CI 0.93-7.94, *P*=.09). Baseline SBPs were comparable in these two groups: 128.4 mm Hg (95% CI 124.8-132.1 mm Hg, n=47) for patients with less than 80% response rate; 130.9 mm Hg (95% CI 128.2-133.6 mm Hg, n=58) for patients with greater or equal to 80% response rate; delta SBP at baseline was 2.5 mm Hg (*P*=.29).

**Figure 2:**
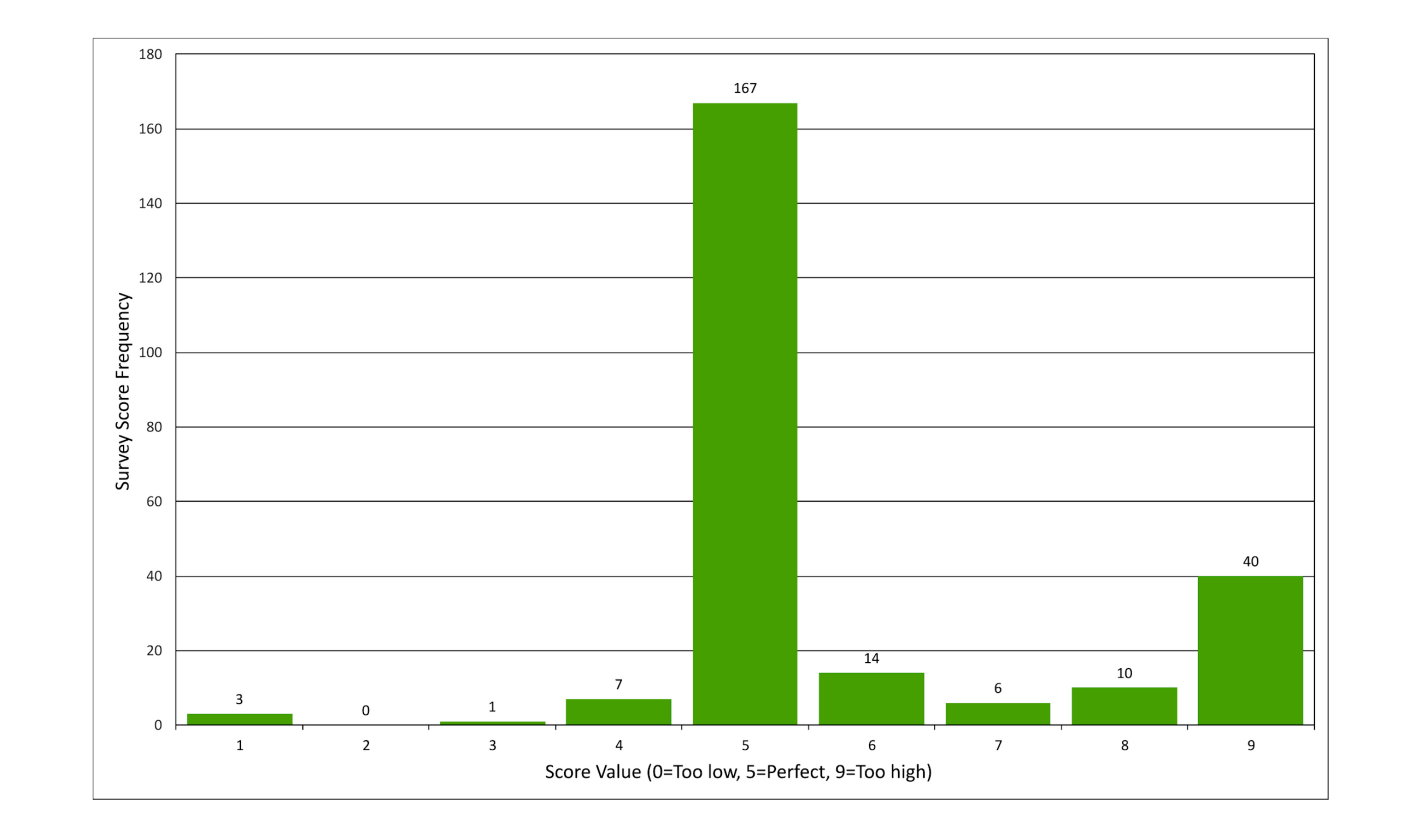
Patient satisfaction with message frequency.

Patients reported an average satisfaction of 8.7 out of 9 (Likert-type scale, 9 is the maximum score) with the service. In a separate question from the same survey assessing patient satisfaction with message frequency, the majority of patient responses (67.3%, 167/248 survey responses ) reported the frequency of messages as “perfect” (Figure 2).

### Improvements in Blood Pressure

The average baseline BP for all patients completing 12 weeks was approximately 129.8/76.4 mm Hg. By the end of the evaluation period, SBP reduced by 10.8 mm Hg (95% CI −14.5 to −7.2) and DBP reduced by 6.6 mm Hg (95% CI −9.9 to −3.4) in the group with baseline SBP of 140 mm Hg or higher, whereas BP for all patients and in the lower baseline SBP group did not change significantly (Table 1).

**Table 1:** Comparison of average blood pressure (BP) at baseline and at weeks 11-12 for the 105 patients who completed week 12 analysis. Significant decreases in systolic BP (SBP) and diastolic BP were found in patients with baseline SBP greater than/equal to 140 mm Hg.

Blood pressure description		Baseline mean	Weeks 11-12 mean	Difference	95% CI	*P* value
**Baseline SBP^a^≥140 (n=22)**						
	SBP	147.3	136.4	−10.8	−14.5 to −7.2	<.001
	DBP^b^	82.4	75.8	−6.6	−9.9 to −3.4	.002
**Baseline SBP<140 (n=83)**						
	SBP	125.2	126.1	1.0	−1.1 to 3.1	.35
	DBP	74.8	74.8	0.08	−1.6 to 1.8	.90
**All patients (n=105)**						
	SBP	129.8	128.3	−1.5	−3.5 to 0.5	.15
	DBP	76.4	75.0	−1.3	−2.8 to 0.1	.06

^a^SBP: systolic blood pressure.

^b^DBP: diastolic blood pressure.

**Figure 3:**
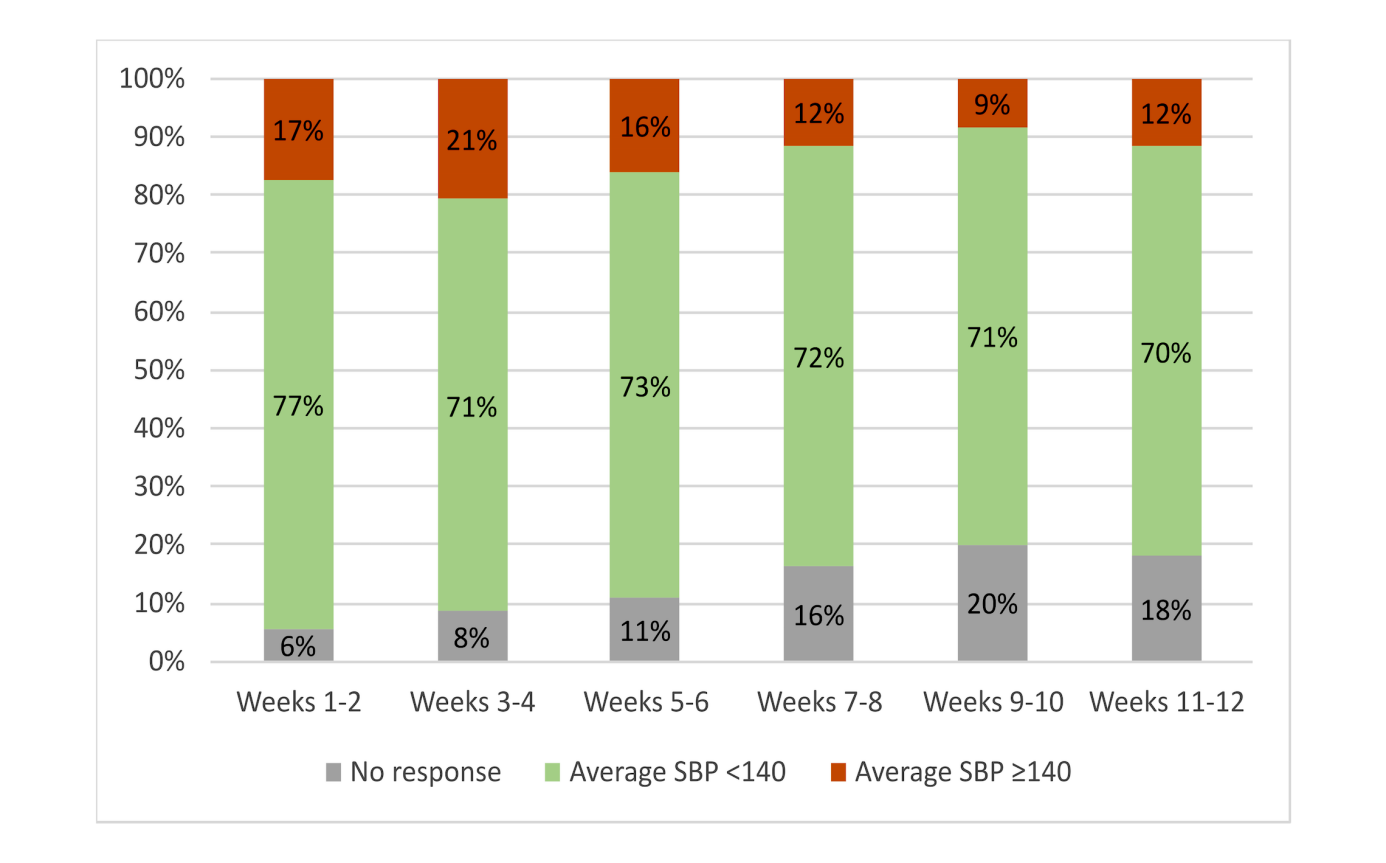
Change in self-reported bimonthly average systolic blood pressures (SBPs) for all patients. No response indicates patients who did not report any BPs during the 2-week period.

**Figure 4:**
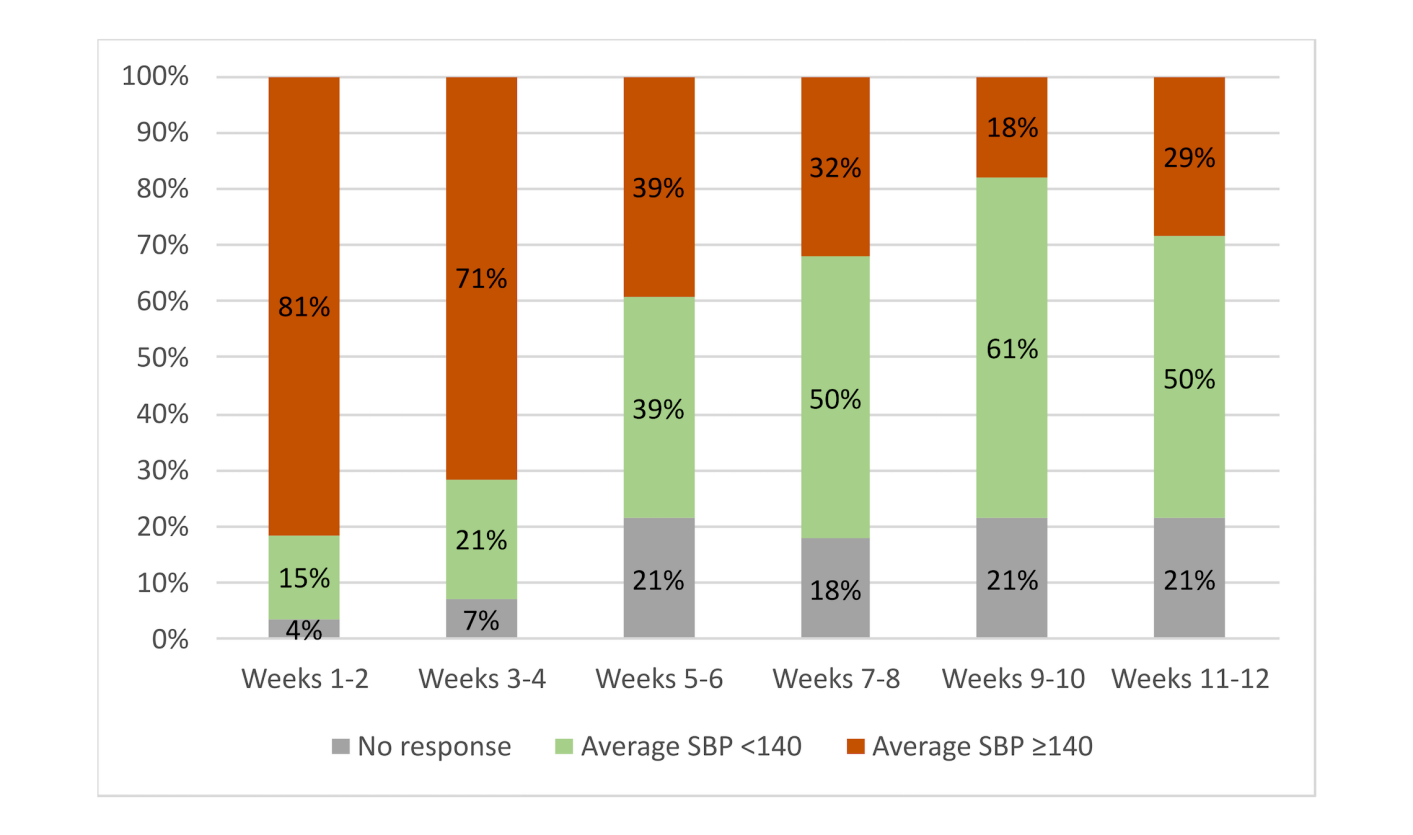
Change in self-reported bimonthly average systolic blood pressures (SBPs) for patients with a baseline SBP greater than or equal to 140 mm Hg. Within the subpopulation of patients with a baseline greater than or equal to 140 mm Hg, there is a significant decline in the proportion of reported SBP averages greater than/equal to 140 mm Hg. The 44 enrolled patients who did not complete baseline analysis and illogical self-reported BPs because of SMS formatting discrepancies were excluded from the analysis. No response indicates patients who did not report any BPs during the 2-week period.

The proportion of all patients reporting SBP greater than/equal to 140 mm Hg each week showed a steady decline over the 12 weeks (Figure 3). Of the 28 patients whose baseline SBP was 140 mmHg or higher, 22 (79%) patients provided sufficient data for analysis at 12 weeks, where 14 out of 22 (64%) reported a final bimonthly average SBP less than 140 mm Hg (Figure 4).

## Discussion

### Response Rate

In this feasibility study, the main outcome is the acceptance of the system, which can be analyzed via response rate and patient satisfaction. Throughout the study, the overall response rate declined slightly (Figure 3); however, among those who responded to the system, 43.8% (57/130) of patients had response rates higher than 80% (data not shown), which suggests the acceptability of the system. We incorporated differential messaging frequency based on level of BP control (2-week mean) where patients who were reporting consistently elevated BP received messages more frequently. However, patients with baseline SBP greater than or equal to 140 mm Hg did not have significantly lower response rates, despite the higher message frequency. The majority of patients in the satisfaction surveys indicated the message frequency to be “perfect,” further supporting the acceptability of this system. Thus, EpxHypertension was uniquely able to monitor BP regularly while maintaining patient satisfaction and a high response rate using the smart scheduling system.

Of note, a higher proportion of patients with greater than 80% response rates achieved mean SBP values below 140 mm Hg when compared with all study participants, thereby suggesting that patients who were more engaged with the intervention have better outcomes.

### Improvements in Blood Pressure

Over the 12-week study period, patients with baseline SBP of 140 mm Hg or higher showed significant reduction in self-reported BP values. Although the overall BP change for all patients was not significant, most patients in the study did not have a baseline BP exceeding 140 mm Hg. Our results demonstrate the value of this intervention for patients who struggle most with BP control and who are subsequently at a higher risk for complications. Whereas a similar phone-based intervention demonstrated effectiveness in reducing BP [24], there have been no validated phone call and text messaging platforms that have demonstrated a significant reduction [25]. We attribute the effectiveness of this system to the unique bidirectional messaging model that allows more active monitoring by patients’ health care teams while simultaneously increasing patient investment in self-health. There is demonstrated stability for patients with baseline SBP less than 140 mm Hg as expected. For patients with an SBP greater than or equal to 140 mm Hg, we demonstrate the ability to drive patients to lower their BP.

### Feasibility in an Outpatient Setting

Our findings arise from the use of EpxHypertension as part of routine clinical practice without additional novel equipment or staff, which demonstrates the applicability and utility of this system in the management of HTN in the standard outpatient setting. Figure 2 presents a favorable scenario in the application of the system in a real-world setting while factoring in likely response rates. As mentioned in the Methods section, the content of the messages were left to the discretion of the providers, which helps demonstrate the external validity of our study and therefore the feasibility of our intervention.

The system has additional benefits of being accessible to patients who may be at a socioeconomically disadvantage, by using user-friendly language that takes into account variability in health literacy and offering free-to-patient text messages and calls. Our intervention aims to facilitate collaborative patient-provider relationships, which have been shown to improve medication adherence in vulnerable, low-income populations [26]. Additionally, poorly controlled HTN is correlated with increased physician visits [27]. We believe EpxHypertension improves patient-provider communication and has the potential to bring HTN under control, which can have a beneficial impact in decreasing the number of in-office visits while placing little additional burden to the health network infrastructure. Reduced in-office visits would be economically beneficial for low-income patients because of fewer missed work hours and fewer transportation costs. Our data demonstrate that patients are willing and able to use nonlinked BP cuffs in combination with EpxHypertension to improve the management of BP.

### Scalability and Reach

Whereas there have been a multitude of telemedicine interventions studied for BP management in hypertensive patients [12,28], EpxHypertension is uniquely beneficial because of its low intensity that will allow large-scale implementation of this intervention. Few telemedicine interventions for chronic disease management in the past decade have taken into account cost in their analysis [28]. Many of these studies provide participants with devices such as BP monitors or smartphones free of charge, which, in addition to increasing overhead costs, also require additional time for installation and participant training. By being able to use any BP cuff and any landline or cell phone, we demonstrate that this system has a potentially higher reach in the populations (ie, lower socioeconomic status [SES] and elderly populations) than those systems described previously. Considering that 40.8% (71/174) of our patients preferred phone calls to SMS text messaging, we believe that the option of using phone calls is appreciated and more convenient than SMS text messaging for a large portion of the patient population at the community clinic.

### Study Limitations and Future Directions

EpxHypertension was designed to collect data and provide clinically important information to providers in a timely manner, thereby allowing them to intervene per their judgement. As such, we have not delineated specifically how providers responded to this new information. Providers may have changed medications or patients may have changed their lifestyle in ways that were not assessed. Baseline demographic data were not collected for this study of community patients. Furthermore, it is possible that the effectiveness of mHealth interventions could vary with a patient’s functional status, health literacy, SES, or other demographic factors not captured in this analysis. Additionally, all data were self-reported, and some measurements could theoretically be fabricated [29], yet the inaccuracy rate was shown to be as low as 16% in underserved patients using a telemedicine intervention [30] and may not be clinically significant. We allowed patients to use their own home BP monitors without additional training, which introduces inherent variability in values recorded and which we recognize as a limitation, although this is more consistent with practical clinical implementation. Most importantly, there was no standard-of-care comparison group in this prospective cohort study. Future studies should therefore evaluate the effectiveness of this intervention with a prospective randomized-controlled trial to test the validity of results presented in this study and to measure additional outcomes such as the total number of in-office visits and medication changes resulting from improved monitoring of BP in patients.

### Conclusions

Epharmix’s EpxHypertension, a bidirectional automated phone and text messaging service, is well accepted by both patients and providers in a community clinic setting based on response rate and patient satisfaction survey results. It has demonstrated feasibility by helping higher-risk hypertensive patients (ie, higher baseline BPs) achieve capturable BP reductions. This cost-effective and widely accessible intervention is a promising new tool for the management of hypertensive patients, especially in the outpatient setting. Future clinical trials are needed to test efficacy and confirm the effectiveness of this intervention in controlling patients’ BPs, as compared with standard of care.

## Abbreviations

AHA: American Heart Association
BP: blood pressure
DBP: diastolic blood pressure
HTN: hypertension
ICD: International Statistical Classification of Diseases and Related Health Problems
mHealth: mobile health
SBP: systolic blood pressure
SES: socioeconomic status
SMS: short message service
